# Reviewers in 2021

**DOI:** 10.1098/rsif.2022.0107

**Published:** 2022-03-16

**Authors:** Richard Cogdell

**Affiliations:** *Editor-in-Chief*

I thank all those referees who helped assess submissions to *Journal of the Royal Society Interface* (volume 18) in 2021. The editorial team very much appreciates the time and dedication given by academics to the peer review process and to recognize their efforts. From previous feedback, we know that most reviewers value recognition of their efforts by the journal and through services, such as Publons, which is integrated with *J. R. Soc. Interface*.

To provide an opportunity for recognition, we list the names of all reviewers who provided reports last year (unless they have opted out or not responded). This article is made permanent and citable by its digital object identifier (DOI). We encourage you to quote your contribution in your applications for tenure and grants or other forms of research assessment. Where you have provided it, we have included your ORCID, so your contribution can be unambiguously assigned to you.

The team really does appreciate all the work our reviewers do on behalf of the journal, and we look forward to working with you again in 2022.

Abdellatif A
ABOU B
Adekunle A
Adler F
Adrakey HK
Adroit B https://orcid.org/0000-0003-3693-9581
Ahluwalia A
Ahmad M
Akkaynak D https://orcid.org/0000-0002-6269-4795
Albert J
Alessandretti L
Allen CN
Althouse B https://orcid.org/0000-0002-5464-654X
Altshuler D https://orcid.org/0000-0002-1364-3617
Ambrose M
Amthor J https://orcid.org/0000-0001-8601-403X
Anderson P https://orcid.org/0000-0001-7133-8322
Andraud M
Aono H https://orcid.org/0000-0003-2427-0833
Apolloni A
Archer S https://orcid.org/0000-0002-1476-607X
Ariel G
Armentano I
Arruda E https://orcid.org/0000-0002-9835-352X
Asabere SB
Asejczyk-Widlicka M
Ashcroft P https://orcid.org/0000-0003-4067-7692
Ashton-Miller J
Asllani M
Atlasi Y
Audo D
Avgar T https://orcid.org/0000-0002-8764-6976
Avital E
Avril S
Azadani AN
Bablani L
Baik L
Baines C https://orcid.org/0000-0002-6918-3648
Bajardi P https://orcid.org/0000-0001-8865-4495
Bakka H
Balle F
Ballester PJ
Banerjee A
Banga J
Barakat A https://orcid.org/0000-0002-7551-3631
Barakat J https://orcid.org/0000-0001-7761-3874
Barker B
Barkoulas M
Bar-On B https://orcid.org/0000-0003-3153-5879
Barrat A https://orcid.org/0000-0001-8683-269X
Barreaux A
Barthelemy M https://orcid.org/0000-0003-0890-9713
Basanta D https://orcid.org/0000-0002-8527-0776
Basdogan C https://orcid.org/0000-0002-6382-7334
Bassett J
Bauer U
Baum D https://orcid.org/0000-0001-8334-6311
Baumgartner W
Bayer C https://orcid.org/0000-0003-0947-6892
Bayly PV https://orcid.org/0000-0003-4303-0704
Beckman R
Beggs C https://orcid.org/0000-0002-6460-9937
Ben Ayed S
Bernard S
Berret B https://orcid.org/0000-0002-0779-7724
Bersi M https://orcid.org/0000-0003-1848-4165
Bertaglia G https://orcid.org/0000-0002-2874-9588
Bertram R
Betta M
Bhagat R https://orcid.org/0000-0002-8928-4534
Bhandawat V
Bhattacharya K https://orcid.org/0000-0002-4943-3814
Bhattacharya S
Bhavnani D
Bianchi M
Biase F
Biewener A https://orcid.org/0000-0003-3303-8737
Bikson M
Bilewicz R
Birnbaum M
Bishop P
Bisin A
Björnham O
Blamires S https://orcid.org/0000-0001-5953-3723
Bluestein D
Blüthgen N https://orcid.org/0000-0002-0171-7447
Bobich E
Boccaccini A
Boga C
Bok M https://orcid.org/0000-0002-3169-8523
Bokil V
Bond T
Boos M
Bootsma M
Borowska A
Borriello E
Bottolo L
Boudinot BE
Bowers DT https://orcid.org/0000-0003-0199-7714
Bradhurst R
Brahmachari S
Brasseur J https://orcid.org/0000-0002-3007-4276
Braun D https://orcid.org/0000-0002-7300-2635
Brazier F
Britton T https://orcid.org/0000-0002-9228-7357
Brook C https://orcid.org/0000-0003-4276-073X
Brown J
Brown N http://orcid.org/0000-0003-3590-0538
Brown R https://orcid.org/0000-0001-6926-4184
Brown S
Bruegger S https://orcid.org/0000-0003-4188-2276
Bruijn S https://orcid.org/0000-0003-0290-2131
Brumley D
Brunnschweiler J
Brusadelli E
Bucchi A
BUENO M-Ange https://orcid.org/0000-0001-5067-217X
Buffet P
Burg D https://orcid.org/0000-0002-0941-3827
Burris D
Bury T
Büscher T https://orcid.org/0000-0003-0639-4699
Butail S
Cadiou H
Caetano-Anolles G
Calder W https://orcid.org/0000-0002-8923-1803
Calovi D https://orcid.org/0000-0002-2452-9801
Calovi D
Campanale J
Cantu C https://orcid.org/0000-0003-1547-5415
Cao H
Cao Z https://orcid.org/0000-0003-2600-5806
Capistrán M
Cappelletti D https://orcid.org/0000-0003-4259-2772
Carbone G
Carichino L https://orcid.org/0000-0002-3891-652X
Carletti T https://orcid.org/0000-0003-2596-4503
Carmigniani R https://orcid.org/0000-0001-6456-9624
Carpenter S
Carrascal L
Carré M
Casademunt J
Casas J https://orcid.org/0000-0003-1666-295X
Caseli L
Cassani S
CatanachT
Cattafesta L
Cerra MC https://orcid.org/0000-0002-2091-928X
Cervo R https://orcid.org/0000-0002-0405-6402
Chacón J
Challenger J
Challis J
Champredon D https://orcid.org/0000-0002-7090-8757
Chan HS
Chang S
Changho K
Chao YHC https://orcid.org/0000-0002-2974-0403
Chateauminois A https://orcid.org/0000-0001-9965-0254
Chen D
Chen F
Chen J
Chen W
Chen Y-G
Cheng B https://orcid.org/0000-0002-6982-0811
Cheong KH https://orcid.org/0000-0002-4475-5451
Cho C
Christofferson R https://orcid.org/0000-0003-2806-1131
Christos N
Ciaccio E
Cinelli M
Cinquemani E
Ciocanel M-V
Civalek Ö
Claessen B
Clapham H
Clausen-Schaumann H
Clemente C https://orcid.org/0000-0001-8174-3890
Cobb R
Codd J https://orcid.org/0000-0003-0211-1786
Codding B https://orcid.org/0000-0001-7977-8568
Colaneri P
Colgate JE
Colubri A https://orcid.org/0000-0001-5559-9661
Comas-Garcia M
Comley J https://orcid.org/0000-0002-3043-7022
Conn A
Conway J https://orcid.org/0000-0003-1474-4230
Cook A
Cooke M https://orcid.org/0000-0001-5254-7559
Coombs D https://orcid.org/0000-0002-8038-6278
Cooper J
Cooper K
CORNUAULT P-H
Corominas-Murtra B https://orcid.org/0000-0001-9806-5643
Cortiços ND https://orcid.org/0000-0002-9446-6199
Coskun U https://orcid.org/0000-0002-5388-5362
Costantini A
Cova TJ
Covington J
Cox S https://orcid.org/0000-0002-9704-0716
Craig M
Crall J
Crandell K https://orcid.org/0000-0002-2150-3198
Crews S
Croft S
Cronin N
Crosato E
Cruz A
Culha M
Czuppon P https://orcid.org/0000-0003-1462-7237
da Silva Rivas GB
Dai X
D'Alba L https://orcid.org/0000-0002-2478-3455
Dalchau N https://orcid.org/0000-0002-4872-6914
d'Alessandro J https://orcid.org/0000-0002-1585-3255
Damette O
Daniel T https://orcid.org/0000-0002-5706-1096
Darlington A
Das Gupta D
Davenport M
Davidson J https://orcid.org/0000-0001-9206-085X
Davis C
De Corato M
de Jong H https://orcid.org/0000-0002-2226-650X
de Kat R
De Luca P
De Nisco G
De Nys H
de Swart R
De Vargas Roditi L
Degen J
DeGregori J
Delgado Chaves F
Delgado-Eckert E https://orcid.org/0000-0001-6415-4971
Delhaye B
Demšar J
den Toonder JMJ
Deng Y
Dennison C
Deravi L
Devetak D
Dewar A
Dhamala M
di Bernardo M https://orcid.org/0000-0002-2171-4745
Di Patti F
Diambra L https://orcid.org/0000-0001-8052-4880
Dickhout J
Ditlevsen S https://orcid.org/0000-0002-1998-2783
Diuk-Wasser M https://orcid.org/0000-0001-8809-3050
Dixon J
Dobrovolny H https://orcid.org/0000-0003-3592-6770
Dokter A
Doyle S https://orcid.org/0000-0003-1679-3247
Doyon J https://orcid.org/0000-0002-3904-7207
Drikakis D
Du J https://orcid.org/0000-0002-8146-9286
Duan H https://orcid.org/0000-0003-1478-5649
Durand B https://orcid.org/0000-0003-0669-1394
Dusek J
Dushoff J https://orcid.org/0000-0003-0506-4794
Dytham C
Edwards J
Edwards J
Eisaguirre J https://orcid.org/0000-0002-0450-8472
Elad D https://orcid.org/0000-0001-8367-6193
Elgar M https://orcid.org/0000-0003-0861-6064
Eliason C https://orcid.org/0000-0002-8426-0373
Elsayad K
Elson E
Endler J https://orcid.org/0000-0002-7557-7627
Engblom S https://orcid.org/0000-0002-3614-1732
Engels T
Equils O
Erdel F
Eren M
Errington T https://orcid.org/0000-0002-4959-5143
Erskine W
Estevez Torres A
Etheridge B
Evans A
Evans S
Ewing D https://orcid.org/0000-0002-9974-8179
Fabbri A
Fadai N https://orcid.org/0000-0001-7717-5421
Fadikar A https://orcid.org/0000-0001-7396-0350
Fakhrullin R https://orcid.org/0000-0003-2015-7649
Falcon-Cortes A
Falcucci G https://orcid.org/0000-0001-6446-4697
Falk D
Famaey N https://orcid.org/0000-0002-7374-8912
Fang Y https://orcid.org/0000-0001-6624-9720
Farji-Brener A https://orcid.org/0000-0001-7251-3866
Farrell A
Fasihi Harandi M
Favretti M
Fedorec A https://orcid.org/0000-0003-0165-1705
Fernandez Messina LM
Ferrari N
ffrench-Constant C
Fieberg J
Fiedler T
Fitzsimons C https://orcid.org/0000-0002-1556-2650
Flack A
Floreano D
Forde J
Forterre Y
Fortini LB
Fossette S
Fraiberger S
Franch-Pardo I https://orcid.org/0000-0003-4346-8757
Franco E
Frank-Ito D
Franklin A https://orcid.org/0000-0002-8650-8344
Franklin D
Franks N https://orcid.org/0000-0001-8139-9604
Freeland S https://orcid.org/0000-0003-4606-7617
Frenkel-Pinter M
Fricks J https://orcid.org/0000-0002-9874-7107
Frigessi A
Fromen C
Funk S https://orcid.org/0000-0002-2842-3406
Fusco D
Fyhrie D
Gabora L https://orcid.org/0000-0002-0382-7711
Gajardo P
Gallo D https://orcid.org/0000-0002-7409-7111
Galvez-Cancino F
Gambaruto A
Ganghoffer J-F
Ganusov V
Garcia G
Garcia-Aznar J
Gardner E
Garza DR
Gaston K
Gautron J
Gauvin L
Gaythorpe K
Geard N https://orcid.org/0000-0003-0069-2281
Gee M https://orcid.org/0000-0001-9293-7201
Gelens L
George EA
Gerba C
Ghatak A
Ghosh I
Ghusinga K
Giannoula A
Gil JP
Gil M
Gilet T https://orcid.org/0000-0002-7339-7796
Gilmour K
Gizzi A https://orcid.org/0000-0001-5350-8156
Glasser J https://orcid.org/0000-0003-3451-5849
Glassmaker N
Gleason R https://orcid.org/0000-0002-6357-4955
Godoy-Diana R https://orcid.org/0000-0001-9561-2699
Goffard P-O
Gogarten J https://orcid.org/0000-0003-1889-4113
Goldman D
Goldwyn E
Gompper G
Goni-Moreno A https://orcid.org/0000-0002-2097-2507
González-Espinoza A
Gonzalez-Parra G https://orcid.org/0000-0001-5847-678X
Gonze D
Gordon A
Gordon M
Gov N https://orcid.org/0000-0001-7774-1139
Govedich F
Graham J
Graner F https://orcid.org/0000-0002-4766-3579
Grebenkov D
Grebogi C
Greenhalgh S https://orcid.org/0000-0003-2484-4147
Gregorič M
Grillet ME
Grima R https://orcid.org/0000-0002-1266-8169
Gros C https://orcid.org/0000-0002-2126-0843
Gross T https://orcid.org/0000-0002-1356-6690
Groundwater PW
Grzech-Leśniak K
Guasto J https://orcid.org/0000-0001-8737-8767
Gubbins S https://orcid.org/0000-0003-0538-4173
Guerrero P
Günther M https://orcid.org/0000-0002-5729-1190
Guo W https://orcid.org/0000-0003-3524-3953
Gupta A
Gurka R https://orcid.org/0000-0002-8907-6663
Gutmann Roberts C
Haghani M https://orcid.org/0000-0003-3904-5196
Han M-W
Han TA https://orcid.org/0000-0002-3095-7714
Hanley Q
Hansen E
Harless W
Harries K
Harrington M
Hartmann M https://orcid.org/0000-0003-0783-1483
Hasan MK
Hassan K
Hattaf K
Haug J https://orcid.org/0000-0001-8254-8472
Haven E https://orcid.org/0000-0003-3852-0194
Hawkes E https://orcid.org/0000-0002-0420-5025
He D https://orcid.org/0000-0003-3253-654X
Hedley R
Heffernan J https://orcid.org/0000-0001-9502-1688
Helbing D https://orcid.org/0000-0002-9898-0101
Hellweger F
Hemelrijk C https://orcid.org/0000-0001-6160-077X
Hendy S https://orcid.org/0000-0003-3468-6517
Henley M
Hens N https://orcid.org/0000-0003-1881-0637
Hensel R
Herzlinger G
Herzog S https://orcid.org/0000-0002-5373-5866
Higginson A https://orcid.org/0000-0002-2530-0793
Higuchi T https://orcid.org/0000-0001-7324-5752
Hilfinger A https://orcid.org/0000-0002-2411-6775
Hill E https://orcid.org/0000-0002-2992-2004
Hilton J https://orcid.org/0000-0002-2787-3827
Hladish TJ
Hladky S
Hoelscher H https://orcid.org/0000-0002-1033-1669
Hogan S https://orcid.org/0000-0001-6012-6527
Hogg R
Hoi H https://orcid.org/0000-0002-3038-9673
Hoinville T https://orcid.org/0000-0001-9073-4515
Holder C https://orcid.org/0000-0002-9149-8996
Holland R https://orcid.org/0000-0003-4495-8061
Holle A
Hollenbeck J
Holmlund P
Holten-Andersen N
Holton G https://orcid.org/0000-0002-9346-1572
Holzner M
Hong Z
Horný L
Hosoda N https://orcid.org/0000-0002-7440-4927
Hossain M https://orcid.org/0000-0002-3338-4173
Hoyle R
Hsu M-C
Hu D https://orcid.org/0000-0002-0017-7303
Hu K
Huang X
Huang Z
Huera-Huarte F https://orcid.org/0000-0002-5447-3183
Hueter B
Hug F https://orcid.org/0000-0002-6432-558X
Hughes T https://orcid.org/0000-0002-5713-9738
Hummel F
Hunt E https://orcid.org/0000-0002-9647-124X
Hunter G https://orcid.org/0000-0001-7022-9482
Huo Y https://orcid.org/0000-0003-4121-3224
Huppertz B
Hurford A https://orcid.org/0000-0001-6461-5686
Hurtado D
Hutchins A
Ibuka Y https://orcid.org/0000-0001-5505-8988
Imhof L
Ingalls B
Iritani R https://orcid.org/0000-0002-2396-1109
Irving A https://orcid.org/0000-0002-0196-1570
Izeta A
Jackson J
Jafari H
Jafarnejad M https://orcid.org/0000-0002-1550-0011
James J
Jana S
Jankauski M https://orcid.org/0000-0001-6305-0564
János T
Jasra A
Jawaid M
Jenner A
Jennings R
Jensen P
Johnson P https://orcid.org/0000-0001-6087-7064
Jolles J https://orcid.org/0000-0001-9905-2633
Jolly MK
Jonas A
Jones A
Jones J
Jones R
Jong ICde
Joseph J
Josic K
Junge M
Kahilainen K
Kain M https://orcid.org/0000-0003-0605-7289
Kalliomäki P
Kamo M
Kamrujjaman M https://orcid.org/0000-0002-4892-745X
Kandail H
Kang C
Kang H-W
Kang V https://orcid.org/0000-0003-0959-1364
Kantor Y
Kao Y-H
Kaplan H https://orcid.org/0000-0002-8583-5714
Kapustin A
Karakostis FA
Kasper R https://orcid.org/0000-0002-3457-1931
Katsikopoulos K
Kattnig D
Katz E
Kaur G
Kavokin K
Ke R https://orcid.org/0000-0001-5307-8934
Kearney R https://orcid.org/0000-0002-5107-6190
Keaveny E https://orcid.org/0000-0001-9103-5687
Kelly S
Kenah E https://orcid.org/0000-0002-7117-7773
Kerr C
Kersh M https://orcid.org/0000-0002-6039-3577
Kershenbaum A
Kier W
Kim BJ
Kim J
King A https://orcid.org/0000-0001-6159-3207
King M
Kissler S
Kivelä M
Kiyono K https://orcid.org/0000-0001-5433-7002
Klein M
Koenderink G
Kollmann B
Kolomenskiy D https://orcid.org/0000-0003-0107-6894
Komarova N https://orcid.org/0000-0003-4876-0343
Kon Kam King G
Kong J https://orcid.org/0000-0002-7557-5672
Korin N https://orcid.org/0000-0001-7244-889X
Kornev K https://orcid.org/0000-0002-4513-1915
Koronfel M
Kraay A
Krishnamurthy A
Kröger R
Kuchenbecker K https://orcid.org/0000-0002-5004-0313
Kumar R
Kung E https://orcid.org/0000-0002-2532-2458
Kunwar A https://orcid.org/0000-0002-8671-7816
Kuo A
Kurvers R https://orcid.org/0000-0002-3460-0392
Kuscu M
Kutsukake N https://orcid.org/0000-0001-8637-6233
Kutz JN https://orcid.org/0000-0002-6004-2275
Kutzke H
La Morgia V https://orcid.org/0000-0001-9915-3194
Labonte D https://orcid.org/0000-0002-1952-8732
Labrosse M
Lahti L https://orcid.org/0000-0001-5537-637X
Lakin M https://orcid.org/0000-0002-8516-4789
Lamb O https://orcid.org/0000-0002-2254-4258
Lambert N
Lancaster J
Langowski J
Larsen PS https://orcid.org/0000-0002-7155-5965
Lassila T
Laurenti L
Laurino M
Lavoie P
Lawson AB
Lazzara G https://orcid.org/0000-0003-1953-5817
Lecheval V https://orcid.org/0000-0001-6041-2718
Leclerc M https://orcid.org/0000-0002-5314-461X
Ledzewicz U
Lee E https://orcid.org/0000-0002-4156-9637
Lee T
Leisnham P
Lejeune E
Lele S
Lenormand M https://orcid.org/0000-0001-6362-3473
Leon T https://orcid.org/0000-0002-7633-677X
Lettinga P
Levin M https://orcid.org/0000-0001-7292-8084
Lewis A
Leys S
Li D
Li H-W
Li L https://orcid.org/0000-0002-6741-9741
Li W
Li Y https://orcid.org/0000-0002-2281-4529
Liao J https://orcid.org/0000-0003-1049-5783
Libby E
Liberson A
Liby K
Lieber RL
Liepe J
Lilienthal A
Lima F
Lin Z
Lion S https://orcid.org/0000-0002-4081-0038
Lipfert J
Lippi V
List C https://orcid.org/0000-0003-1627-800X
Liu H https://orcid.org/0000-0002-8687-3237
Liu J http://orcid.org/0000-0002-7445-3518
Liu L https://orcid.org/0000-0002-5760-2964
Liu X
Liu Y
Loder A
Lohse D
Lou J
Louis A
Lu T
Lugito NPH
Luonan C
Luque A https://orcid.org/0000-0002-5817-4914
Lycett S https://orcid.org/0000-0003-3159-596X
Lythe G
Ma W
Ma Y
Macaskill C
Mackay D
Mackie A
Maden M
Mail M
Makovicky P
Mallory J
Mangal T https://orcid.org/0000-0001-9222-8632
Manheim D
Manley E
Manore C https://orcid.org/0000-0003-2534-9316
Mao H
Marchisio M
Markovitch O https://orcid.org/0000-0002-9706-5323
Marquet P https://orcid.org/0000-0001-6369-9339
Marr L https://orcid.org/0000-0003-3628-6891
Marreiros J
Marshall J https://orcid.org/0000-0001-9006-6713
Martens P
Martin O https://orcid.org/0000-0002-5295-5963
Martin W
Martino D
Masen M
Maslennikov O
Massaroni C
Masson J-B
McGinty S https://orcid.org/0000-0002-2428-2669
McGlade J
McNamara J https://orcid.org/0000-0002-4235-3045
McPhedran R http://orcid.org/0000-0002-1315-141X
Medo M https://orcid.org/0000-0001-8865-9085
Mehrotra R
Mehta R https://orcid.org/0000-0002-6244-9968
Mei L
Meng Q-H
Menzel R
Meron E https://orcid.org/0000-0002-3602-7411
Meyer L
Michaelian K
Michalik P https://orcid.org/0000-0003-2459-9153
Michelin S
Miler K https://orcid.org/0000-0001-7684-0629
Miller K
Milner-White J https://orcid.org/0000-0002-3849-4363
Milton J https://orcid.org/0000-0001-5037-2548
Minati L
Miyamori Y https://orcid.org/0000-0003-2686-8062
Möbius W
Moghadas S https://orcid.org/0000-0003-4414-0227
Momeni B https://orcid.org/0000-0003-1271-5196
Mongeau J-M https://orcid.org/0000-0002-3292-6911
Montresor M
Moore J
Moore S
Moored K
Morel P
Morozov A https://orcid.org/0000-0002-6935-3563
Morozov A
Morris M
Mounce B
Mueller B
Muijres F https://orcid.org/0000-0002-5668-0653
Mukwaya A
Muloi D https://orcid.org/0000-0002-6236-2280
Munasinghe M
Murawaki Y
Murfee W
Murray J
Murray R
Murray RM
Myung J https://orcid.org/0000-0002-2529-8013
Nabawy M https://orcid.org/0000-0002-4252-1635
Nain A
Nakata T
Nandi S
Nangia S
Narayan R
Narsimhan V
Nataraj C
Nax H
Neal P
Ngoepe M https://orcid.org/0000-0002-3639-9063
Ngonghala C https://orcid.org/0000-0002-8441-9495
Nguyen D
Nguyen L
Niepa T
Nikishova A
Nikolic N
Nishi A
Nishizaki M
Niu S
Niven R https://orcid.org/0000-0001-5781-1259
Nonomura T https://orcid.org/0000-0001-7739-7104
Nunes A
Nunes C
Obolski U
O'Brien M https://orcid.org/0000-0001-6433-8295
Ochab-Marcinek A https://orcid.org/0000-0002-2462-0656
O'Clery N
O'Connor P
Oddo CM
O'Dwyer J https://orcid.org/0000-0003-1180-8622
Ohyama T
Okayama K
Okuda S https://orcid.org/0000-0003-3792-8067
Oliveira M https://orcid.org/0000-0003-3407-5361
Olson B
Olsson O
Olufsen M https://orcid.org/0000-0003-2694-0044
O'Neill P
Ong SWX
Opatowski L
Orr C
Orzack S
Osorio D
Ostoja-Starzewski M https://orcid.org/0000-0002-3493-363X
Özden S
Ozkirimli E
Paaijmans K
Paál G
Pagnini G
Palma V
Panasenko G
Papangelo A https://orcid.org/0000-0002-0214-904X
Parisi A https://orcid.org/0000-0003-2972-9770
Park M
Parkinson DB
Parnell A https://orcid.org/0000-0001-8606-8644
Parrish C https://orcid.org/0000-0002-1836-6655
Parslew B https://orcid.org/0000-0002-0070-488X
Passmore J https://orcid.org/0000-0001-7791-3439
Paton R https://orcid.org/0000-0002-4512-9399
Patton A
Paz S
Pedrassoli J
Peixoto P https://orcid.org/0000-0003-2358-3221
Peña J https://orcid.org/0000-0002-4137-1823
Pena-Miller R
Perc M https://orcid.org/0000-0002-3087-541X
Perez-Carrasco R https://orcid.org/0000-0001-5348-8829
Perkins A https://orcid.org/0000-0002-7518-4014
Perrino G https://orcid.org/0000-0001-8095-9139
Perumal A
Peters K
Phillips N
Piarroux R
Piepenbrink K
Pierce C
Pincebourde S
Piontek J
Plank M https://orcid.org/0000-0002-7539-3465
Pleijel H
Pleimling M
Poncelet D
Poon E
Porta A https://orcid.org/0000-0002-6720-9824
Portone T
Potoyan D https://orcid.org/0000-0002-5860-1699
Potvin-Trottier L
Praharaj S
Prakash V
Prete C
Prévost C
Preziosi L
Prieto Curiel R https://orcid.org/0000-0002-0738-2633
Prokop J https://orcid.org/0000-0001-6996-7832
Ptak M
Pullano S
Putman N https://orcid.org/0000-0001-8485-7455
Quinn D https://orcid.org/0000-0001-5880-3728
Rachinskii D
Raghav V https://orcid.org/0000-0001-8667-4409
Raiteri B https://orcid.org/0000-0002-2078-9075
Ramirez Kitchen C
Ramon J
Randler C https://orcid.org/0000-0002-7357-2793
Ratcliff W https://orcid.org/0000-0002-6837-8355
Rauch C
Ravi S https://orcid.org/0000-0001-7397-9713
Rawson F
Rayner M
Rayz V
Read D
Read E https://orcid.org/0000-0002-9186-284X
Read J https://orcid.org/0000-0002-9697-0962
Rebellato L
Rebolledo R
Recker M https://orcid.org/0000-0001-9489-1315
Reddy S
Reid J https://orcid.org/0000-0001-6022-1778
Reif J
Reina A https://orcid.org/0000-0003-4745-992X
Reluga T
Repetto R https://orcid.org/0000-0001-9332-5063
Reveret L
Ribak G
Ribeiro M
Rietkerk M
Roberts M https://orcid.org/0000-0003-2693-5093
Roberts T
Robertson A https://orcid.org/0000-0002-5063-4293
Robinson GE
Rocklov J
Rode C
Rodgers J
Roecker K https://orcid.org/0000-0003-1133-2548
Roka Moiia Y
Roldán-Alzate A
Romenskyy M https://orcid.org/0000-0003-2565-4994
Romero-Severson EO
Romey W http://orcid.org/0000-0003-2759-6791
Römhild R
Rosario M https://orcid.org/0000-0001-9969-6746
Rosas A
Rose C
Rose J
Rossi F https://orcid.org/0000-0002-1854-532X
Rossmann J
Roy A
Rozenblat C
Rozins C https://orcid.org/0000-0003-1503-4871
Rubenstein D https://orcid.org/0000-0002-4999-3723
Rubin J
Ruffoni D https://orcid.org/0000-0003-3714-7594
Ruggeri Z
Rugonyi S https://orcid.org/0000-0001-9262-7959
Runkle J
Russo G
Ruz G
Saal H https://orcid.org/0000-0002-7544-0196
Sacco P https://orcid.org/0000-0002-5559-2889
Safan M
Safran S
Saha D
Saha R
Samaee M
Sambaraju K
Samy A
Sánchez A
Sanderson SL
Sandler S
Sano K https://orcid.org/0000-0002-0839-8549
Santi P
Santon M https://orcid.org/0000-0002-9397-4052
Satake A https://orcid.org/0000-0002-0831-8617
Sauer R https://orcid.org/0000-0001-5625-8295
Sauro H https://orcid.org/0000-0002-3659-6817
Saxena S
Saxena S
Sayakkara A https://orcid.org/0000-0001-9558-7913
Sayan P
Scarabel F
Schäfer M
Schlag K
Schmitt S
Schuler A https://orcid.org/0000-0003-4853-6130
Schumacher L https://orcid.org/0000-0003-0797-3406
Schwager H
Schwarz U
Scoglio C
Seddon A
Semboloni F
Sepulchre R
Seto M https://orcid.org/0000-0003-4709-5206
Shah D
Shahrezaei V
Shain H
Shakiba N
Sharma AD https://orcid.org/0000-0002-8458-9216
Shaw M
Shawkey M https://orcid.org/0000-0002-5131-8209
Shazly T
Sheard C https://orcid.org/0000-0002-8259-1275
Shen Z
Sheng J
Shirokawa Y https://orcid.org/0000-0003-4825-7190
Shnerb N
Shockley K
Shriner S https://orcid.org/0000-0003-0349-7182
Shrivastava A
Shultz J
Sieber J https://orcid.org/0000-0002-9558-1324
Sigaeva T
Sigwart J https://orcid.org/0000-0002-3005-6246
Sih A
Sikdar S https://orcid.org/0000-0001-6897-0247
Silber M
Simpson R
Singh K
Singh N
Singhal N https://orcid.org/0000-0002-9461-688X
Singh-Gryzbon S
Sirl D
Smink A
Smith A
Smith D
Smith L
So M
Soloveichik D
Somarelli J
Somers J
Sonia Petronio A
Sontag E https://orcid.org/0000-0001-8020-5783
Souron A
South L
Søvik E
Sponberg S https://orcid.org/0000-0003-4942-4894
Sprinzak D
Sridharan V
Srinivasan M
Srygley R https://orcid.org/0000-0001-5510-3873
Stadnitski T
Stan G-B
Staniczenko P https://orcid.org/0000-0001-5091-8416
Stark A https://orcid.org/0000-0002-4217-2850
Stawski C
Steel H
Steiner U
Steinmann T https://orcid.org/0000-0002-2013-7747
Stenum J
Stevenson J
Stewart J
Stiehl T https://orcid.org/0000-0001-9686-9197
Stilianakis N
Stok K https://orcid.org/0000-0002-0522-4180
Storozum M
Strandburg-Peshkin A https://orcid.org/0000-0003-2985-6788
Street S https://orcid.org/0000-0001-8939-8016
Stroberg W https://orcid.org/0000-0001-8900-4515
Strömbom D https://orcid.org/0000-0002-9564-2529
Strzelecki M
Stubbs C https://orcid.org/0000-0002-3121-3223
Sturla F https://orcid.org/0000-0001-7317-304X
Suarez A
Sugiyama J
Suhonen H
Sullivan R
Sumpter D https://orcid.org/0000-0002-1436-9103
Sutcliffe M
Suter T
Suzuki R
Svetlichny LS
Swart A
Számadó S
Taha H
Takeishi N https://orcid.org/0000-0003-0111-2269
Tam C
Tamm K https://orcid.org/0000-0002-2455-8258
Tan C
Tan J http://orcid.org/0000-0002-2665-4614
Tanaka M https://orcid.org/0000-0003-2198-1402
Tanaka Y
Tang H
Tang J https://orcid.org/0000-0002-4963-1028
Tannenbaum A
Tauber (né Esser) F https://orcid.org/0000-0001-7225-1472
Taute K
Taylor A
Teaford M
Tennant W https://orcid.org/0000-0001-5822-6887
Testori M https://orcid.org/0000-0001-7292-7129
Thakur K
Thakur N
Thakur S
Thiria B
Thomas A
Thomas M
Thomas P https://orcid.org/0000-0003-4919-8452
Thornley S
Thulke H-H https://orcid.org/0000-0002-7670-2231
Thurow B https://orcid.org/0000-0002-2166-9067
Thybring E
Tiago J https://orcid.org/0000-0001-8979-2136
Tian B
Timerman D https://orcid.org/0000-0002-8772-6271
Tiomkin S
Tizzoni M https://orcid.org/0000-0001-7246-2341
Tjaden N
Tlusty T https://orcid.org/0000-0002-9662-3725
Tobalske B
Tokuriki N
Torii R
Toro-Ibacache V https://orcid.org/0000-0003-2265-8180
Traniello J
Trenti T
Triantafyllou M
Tricou V https://orcid.org/0000-0002-5465-8647
Trounson A
Trusty P
Tsaturyan A
Tsukruk V
Tůma R
Tuomisto J
Tupper P https://orcid.org/0000-0002-4340-4481
Turunen M
Tyson R https://orcid.org/0000-0002-7373-4473
Unicomb S https://orcid.org/0000-0003-4780-6303
Urban J
Usherwood J https://orcid.org/0000-0001-8794-4677
Vallino J
van Baalen M https://orcid.org/0000-0002-3713-5967
van Breugal F
Van Buren T
van den Berg P https://orcid.org/0000-0001-7886-9345
van der Meijden A
van Leeuwen E https://orcid.org/0000-0002-2383-5305
van Leeuwen J
Van Pottelbergh T https://orcid.org/0000-0001-7358-7509
Vande Geest J
Vassen B
Veller C
Venkatramanan S
Verlhac MH
Vicent JF
Villani M
Villaverde A https://orcid.org/0000-0001-7401-7380
vincent J
Virag L
Vizzari G
Voigt D https://orcid.org/0000-0003-2772-8504
Vologodskii A
Volpert V
von der Haar T https://orcid.org/0000-0002-6031-9254
von Seidlein L https://orcid.org/0000-0002-0282-6469
von Walden F
Vorobyev M https://orcid.org/0000-0001-7615-5816
Votta E
Vulin C
Wagner C
Wagner D
Wakefield J
Waldherr S https://orcid.org/0000-0002-0936-579X
Walker I
Walker M https://orcid.org/0000-0001-5119-9118
Wall C
Wallot S
Wan K-T http://orcid.org/0000-0001-8268-6381
Wandelt S
Wang B
Wang J
Wang K
Wang L https://orcid.org/0000-0002-5371-2138
Wang P
Wang R
Wang Z
Warrick D
Waschina S
Waters J https://orcid.org/0000-0001-7077-9441
Watson R
Watton P
Wegst UGK https://orcid.org/0000-0002-9057-415X
Wei J https://orcid.org/0000-0001-8859-8462
Weibull J
Weihmann T https://orcid.org/0000-0002-6628-1816
Weihs D https://orcid.org/0000-0001-8992-6659
Weijer C
Wells K https://orcid.org/0000-0003-0377-2463
Werth A https://orcid.org/0000-0002-7777-478X
West J https://orcid.org/0000-0001-9579-4664
White A
White N
Whittaker D
Wierschem A
Wiesner K https://orcid.org/0000-0003-2944-1988
Wilkinson D https://orcid.org/0000-0002-9560-6499
Williams C https://orcid.org/0000-0002-4758-1506
Williams M https://orcid.org/0000-0002-7284-8014
Williams-Hatala EM
Wilson A
Wilts B https://orcid.org/0000-0002-2727-7128
Winner K https://orcid.org/0000-0002-8838-8905
Wiseman D
Witschey W https://orcid.org/0000-0003-1669-2120
Wodarz D https://orcid.org/0000-0002-8017-3707
Wolf L https://orcid.org/0000-0003-0274-599X
Woodward J
Wu K-Ta
Wu W
Wu Y
Wu ZJ
Wymant C
Xia T
Xu X
Xu Y https://orcid.org/0000-0003-3898-022X
Xu Z
Yakupov T
Yao H http://orcid.org/0000-0003-0549-2246
Yap CH https://orcid.org/0000-0003-2918-3077
Yeh Y-T
Yeung E https://orcid.org/0000-0001-7630-7429
Yin J
Yin L https://orcid.org/0000-0002-0262-0655
Yoon SO
Young J https://orcid.org/0000-0002-8671-990X
Yousefzadeh M https://orcid.org/0000-0001-8644-1939
Youssofzadeh V
Zachreson C https://orcid.org/0000-0002-0578-4049
Zadoks R
Zafeiris A
Zahradka D
Zama F
Zamparo P https://orcid.org/0000-0002-6919-6721
Zartman J
Zee P http://orcid.org/0000-0003-2594-9602
Zhang J https://orcid.org/0000-0001-7107-4814
Zhang J https://orcid.org/0000-0002-6858-9777
Zhang P https://orcid.org/0000-0001-8237-1259
Zhang Y
Zhang Z https://orcid.org/0000-0002-6752-3434
Zhao K
Zhao R
Zhdanov O
Zhishen, GE
Zhu S
Zierenberg J
Zino L https://orcid.org/0000-0002-0946-6523
Zohdi T https://orcid.org/0000-0002-0844-3573
Zohdy S
Zun P https://orcid.org/0000-0001-6176-1143
Zurakowski R https://orcid.org/0000-0003-3726-3114
Zyssett P

